# Patterns of care and outcomes following external ventricular drain placement: Insights from the England HES administrative data set

**DOI:** 10.1016/j.bas.2025.105906

**Published:** 2025-12-16

**Authors:** Daniel Thompson, Adam Wahba, Adam Williams, Peter Hutchinson, Adel Helmy, David Cromwell

**Affiliations:** a Department of Clinical Neurosciences, University of Cambridge, UK; b Department of Neurosurgery, North Bristol NHS Trust, UK; c Leeds Institute of Medical Research, School of Medicine, Worsley Building, University of Leeds, Leeds, LS2 9JT, UK; d Department of Health Services Research and Policy, London School of Tropical Medicine, UK

**Keywords:** External ventricular drain, Performance monitoring, Quality indicators, Risk-adjustment

## Abstract

**Objectives:**

To evaluate the outcomes of patients undergoing external ventricular drain (EVD) insertion in England, focusing on the timing of EVD relative to index neurosurgical procedures, and to assess the implications for benchmarking and performance monitoring between neurosurgical centres.

**Methods:**

We conducted a retrospective cohort study using Hospital Episode Statistics. Adult patients (≥16 years) undergoing EVD insertion between April 2013 and March 2020. Outcomes included 90-day mortality, length of stay (LOS), and emergency readmission within 30 days. Multivariable logistic regression was used for mortality and readmission, with adjustment for age, admission method, comorbidity (RCS Charlson index), and neurosurgical clinical category. A quantile regression model was performed with LOS as the outcome.

**Results:**

The cohort comprised 10,239 patients. Crude 90-day mortality was 26.7 % overall, highest in the EVD-only group (43.2 %) and lowest when EVD was performed with an index procedure (19.7 %). Mortality rose with age, comorbidity, emergency admission, and was highest in Oncology, Vascular, and General & Trauma categories. The final risk-adjustment model showed good discrimination (AUC 0.71) and reduced apparent inter-unit variation in mortality.

**Conclusions:**

Our findings demonstrate that treating all EVD insertions as a single cohort obscures clinically meaningful differences in patient trajectories and leads to misleading comparisons of outcomes. Although the absence of detailed severity markers in administrative data means that conclusions about quality of care must be interpreted cautiously, this study illustrates how carefully constructed, clinically meaningful cohorts can transform the interpretation of common neurosurgical procedures.

## Highlights

• National cohort study of 10,239 patients undergoing EVD insertion in England.
• Mortality (26.7 %) and outcomes varied substantially by EVD timing and Neurosurgical clinical category.
• Distinct care patterns identified for “EVD only” and “EVD after index procedure”.
• Risk-adjusted models and funnel plots demonstrate inter-unit outcome variation.
• Findings support nuanced cohorting of common procedures for audit and benchmarking.

## 1 Introduction

The insertion of external ventricular drains (EVDs) is a commonly performed neurosurgical procedure, often used in the management of raised intracranial pressure and the treatment of cerebrospinal fluid (CSF) infection (Muralidharan, 2015). The outcomes of patients managed using EVD are of interest to the Neurosurgical community given the volume of procedures and the potential for complications (Muralidharan, 2015; Mahto et al., 2023). This also makes the procedure a candidate for monitoring the quality of care received at different Neurosurgical centres since it is easier to reach a statistical significance when evaluating outcomes (Bottle et al., 2017; Kiernan et al., 2022).

EVDs are inserted in the management of a variety of pathologies but there has been a tendency in the literature to treat them as an homogenous group of patients, especially when assessing outcomes. The British Neurosurgical Trainee Research Collaborative (BNTRC) led audit looked into all EVD-related infections and demonstrated an in-hospital infection rate of 9.3 % as well as a 30-day mortality rate of 25.2 % (Jamjoom et al., 2018). Wahba et al. subsequently investigated the patterns and outcomes of Neurosurgery procedures in England and categorised EVD as belonging to the “CSF disorders” clinical category (Wahba et al., 2022a). However, in reality the care bundles EVDs form a part of are diverse, the cohort is heterogenous and accounting for differences in case mix between institutions for performance monitoring is difficult.

In our study, we examined the value of reporting on the patterns of care and short-term outcomes in this patient cohort with respect to the timing of the EVD procedure in relation to an index Neurosurgical procedure, and explored issues with comparing performance between neurosurgery centres given the heterogeneity of the cohort. Patients were categorised as either only undergoing an EVD insertion, undergoing an EVD insertion before an index Neurosurgical procedure, at the same time as an index Neurosurgical procedure, or subsequent to an index Neurosurgical procedure. This approach aimed to capture different patient journeys which were hypothesised to have different patterns of outcomes: having a standalone EVD procedure may equate to a salvage operation, an EVD before an index procedure may denote a class of patients that have been stabilised to proceed to a definitive procedure, an EVD at the same time as an index procedure may be planned to prevent subsequent hydrocephalus and an EVD after an index procedure might be as a complication of the index procedure or a progressive pathology. The study focused on common quality indicators for surgical patients: postoperative mortality, length of stay and emergency readmission within 30 days (Ansari et al., 2018; Donabedian, 1966).

This study was performed using the Hospital Episode Statistics database linked to the Office for National Statistics (ONS) death register as its source of data on patients admitted to NHS neurosurgical centres. The HES dataset is the hospital administrative database for NHS hospitals in England, and allows admissions by individuals to be followed over time (Herbert et al., 2017). The explicit benefits to this are the complete capture of patients that improves the generalisability of findings. It further allows comparison of practice between units and the data is readily available for ongoing surveillance of services. This is an administrative data set that does not include clinical variables such as Glasgow Coma Score. However, operation codes and diagnostic codes as well as patient demographic data mean that if a nuanced approach is used then practice can be compared between centres. Any interpretation of outcome differences must, however, rcognise the absence of clinical parameters and the risk that residual confounding and unique local practice patterns may influence results.

The primary aim of the study was to characterise patterns of care and outcomes for patients undergoing EVD insertion using the England HES data. The study was not designed to test a causal relationships between the timing of EVD insertion and outcomes. Instead, we sought to demonstrate that a data-driven approach to defining and cohorting a common Neurosurgical operation could support future audit and benchmarking work that seeks to evaluate the quality of Neurosurgical care.

## 2 Methods

### 2.1 Data sources and cohort definition

This study was a retrospective cohort study that used the hospital episode statistics (HES) dataset linked to the Office for National Statistics (ONS) death register as its source of data on patients admitted to NHS neurosurgical centres. The HES dataset contains information on the type and timing of admissions, diagnosed conditions and procedures performed, and admissions for an individual are allocated the same identifier so they can be linked. Patient conditions are captured using the International Classification of Diseases, version 10 (ICD-10), and procedures are defined using the UK Office of Population Censuses and Surveys classification (OPCS, version 4)(Herbert et al., 2017).

The study cohort contained all admissions of adults (≥16 years) that involved the insertion of an EVD at one of the 24 adult NHS neurosurgical units in England between April 1, 2013 and March 31, 2020 (seven years). The OPCS-4 code “A201” was used to identify admissions for patients who had undergone EVD insertion. After identifying these admissions, the NNAP Coding Framework was used to allocate an Index neurosurgical procedure (other than an EVD). The Framework groups OPCS codes into three types (cranial, spinal, other) and 12 clinical categories which reflect the neurosurgery subspecialty practice (supplementary appendix 1 & 2). The Index procedure was denoted as the first Neurosurgical procedure within the NNAP coding framework performed within the admission (other than an EVD). Some minor procedures listed in the appendix were omitted (supplementary appendix 3). The Neurosurgical clinical category was based on either the index procedure or (for those patients who only had an EVD procedure) diagnostic codes (ICD-10). This process allowed each admission to be grouped into one of four EVD types: (1) having only an EVD procedure, (2) having an EVD procedure before an index procedure, (3) an EVD on the same date as an index procedure, or (4) an EVD after an index procedure. When a patient had multiple EVD, the category was assigned according to when the first EVD occurred, e.g. if an EVD was performed before and after an index procedure, the patient was allocated to the “before index” category.

### 2.2 Patient variables

The variables defined in the study included: patient demographics (age on admission, sex and socio-economic deprivation), comorbidity index using the Royal College of Surgeons of England Charlson Comorbidity Index (RCS CCI) (Armitage et al., 2010), method of admission (elective or emergency), details of neurosurgical conditions. Area-level socioeconomic deprivation was measured using the Index for Multiple Deprivation (IMD), with the analysis grouping areas into quintiles based on the ranks of their overall IMD values. The RCS CCI was derived by searching for selected ICD-10 codes within the diagnosis fields of HES records of the admission containing the EVD procedure as well as in any admissions during the preceding year.

The outcome measures used were length of stay (LOS), emergency readmission to hospital within 30 days of discharge, and 90-day mortality rates. The date of death was obtained from the ONS Death Registry. Mortality rates were defined from the date of the first Neurosurgical procedure the patient underwent. The LOS was defined as the period of time from the index admission date to discharge from the Neurosurgical centre where they underwent their EVD procedure.

### 2.3 Statistical analysis

The study undertook a complete case analysis. Descriptive statistics summarised the characteristics of the patient cohort. An exploration of patient survival within each EVD category was performed using the Kaplan-Meier method, and extended to 365 days post-procedure.

Multivariable logistic regression was used to investigate the relationship between 90-day postoperative mortality, the timing of the EVD procedure, and patient characteristics, including: age on admission, sex, admission method (elective or emergency), deprivation quintile, Neurosurgical clinical category, and the RCS CCI. Age was included in the model as a continuous variable. Interactions between explanatory variables were examined and those which improved the Bayesian Information Criterion (BIC) are included in Table 2. Given the likelihood of EVD timing varying according to admission type or Neurosurgical clinical category, interaction terms were systematically tested. Specifically:

• Age x elective admission
• EVD timing x admission type
• EVD timing x Neurosurgical category

Sex and deprivation status were not found to improve the model fit using the “testparm” function in STATA and therefore were eliminated from the final model. Logistic regression was also used to model how patient factors were associated with 30-day emergency readmission rates. The same factors as were used to predict 30-day mortality were once again explored. The performance of the models was evaluated by calculating their calibration and discrimination (Alba et al., 2017). Sex, RCS Charlson score, deprivation status and admission type did not improve the model and therefore were eliminated from the final risk adjustment.

Indirect standardisation was used to produce risk adjusted outcomes for the NHS neurosurgery units, with the logistic regression models being used to estimate expected number of events (postoperative deaths, readmissions) at each organisation given their casemix. Funnel plots for the crude and risk adjusted outcomes were used to show how risk adjustment changed the variation between organisations, and the identification of units whose performance fell outside the expected range. This was indicated by 95 % and 99.8 % control limits plotted around the overall mean rate. Units with values outside these control limits are considered statistical outliers, suggesting that their outcomes differed from the national average beyond what can be attributed to chance variation.

To examine the median LOS across units, we fitted a quantile regression model with LOS as the outcome and unit as a categorical variable. Explanatory variables of sex, deprivation status, admission type, EVD timing, RCS CCI and Neurosurgical clinical category were all used in this model. Median values and their 95 % confidence intervals were then estimated as marginal rates. Wider confidence intervals are found for units performing fewer procedures. The adjusted caterpillar plot utilised a risk adjustment model that included all previously mentioned variables as well as unit as a categorical variable. Data analysis was performed using Stata, version 17 (StataCorp LP, College Station, TX).

## 3 Results

The study cohort contained 10,239 patients who had at least one EVD procedure between April 1, 2013 and March 31, 2020 in an English NHS neurosurgery unit. There were 2954 patients who had only EVD insertion, 2227 with an EVD inserted prior to another index procedure, 3425 with an EVD inserted on the same day as another index procedure and 1633 where an EVD was inserted after an initial Neurosurgical index procedure.

The average age of patients in the overall cohort was 54.4 years, and 48.6 % were male. The majority of patients had a low comorbidity burden, with 53 % of patients having an RCS CCI of 0. The most prevalent Neurosurgical clinical categories were General & Trauma (30.2 %), CSF disorders (17.3 %) and Neurovascular surgery (39.2 %).

There were some notable differences in the demographic characteristics of patients within the EVD categories (Supplementary Table 1). A greater proportion of patients who had EVD only were older (23.4 % over 70 years old), male (57.1 %) and from the General & Trauma groups (58.1 %). The common clinical categories for patients having an EVD before an index procedure were CSF disorders or Neurovascular clinical category (26.4 % and 57.4 % respectively). Patients having EVDs inserted on the same day as an index procedure or after, were spread across the CSF disorder (18 % and 20 %), vascular (36 % and 37 %), and General & Trauma categories (26 % and 19 %).

**Fig. 1:**
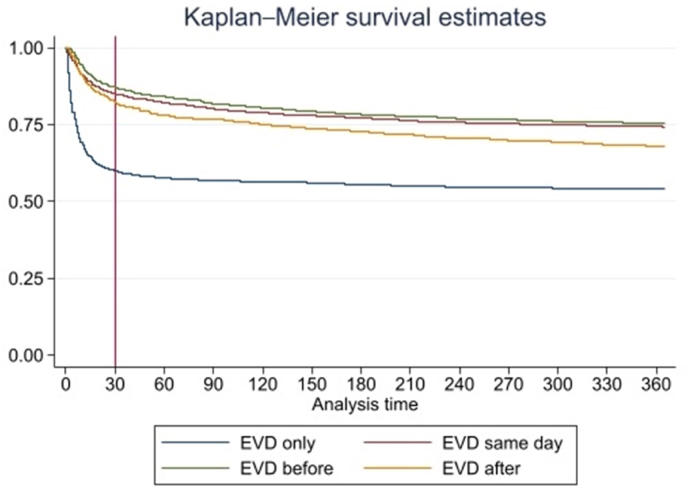
Kaplan-Meier survival curve comparing survival rates for the different EVD categories over the first year post-insertion.

The crude 90-day mortality rate for the overall cohort was 26.7 %. The mortality rate was higher among older patients and as the number of comorbidities increased (see Table 1). The EVD-only category had a significantly higher 90-day mortality rate overall (43.2 %) than any other EVD category (see Supplementary Table 1), with the majority of deaths occurring within 30 days of the procedure. The risk within 30 days was lower for the other EVD categories procedures. However, the risk of death flattens more quickly over the subsequent period for the EVD only category compared to the other three groups (Fig. 1).

**Table 1:** Descriptive statistics for Cranial patients and the individual NNAP Clinical Categories.

		No. of patients		90-day mortality
**Total**		10,239	(%)	26.7 %
**Age at admission (years)**				
	18–39	1936	(18.9)	17.1 %
	40–49	1654	(16.2)	24.7 %
	50–59	2374	(23.2)	24.7 %
	60–69	2281	(22.3)	29.7 %
	70 +	1994	(19.5)	36.5 %
**Sex**				
	Female	5253	(51.3)	26.4 %
	Male	4985	(48.6)	26.9 %
**Deprivation**				
	1 (Most)	2353	(22.9)	26.8 %
	2	2149	(20.9)	28.5 %
	3	1931	(18.8)	25.4 %
	4	1922	(18.7)	26.4 %
	5 (Least)	1883	(18.3)	26.0 %
**Admission type**				
	Non-elective	9324	(91.0)	27.8 %
	Elective	914	(8.9)	15.5 %
**RCS Charlson Comorbidity Score**				
	0	5286	(51.6)	23.3 %
	1	3096	(30.2)	26.0 %
	2	1243	(12.1)	34.8 %
	3	619	(6.0)	43.2 %
**EVD category**				
	EVD only	2954	(28.8)	43.2 %
	EVD before index procedure	2227	(21.8)	18.3 %
	EVD with index procedure	3425	(33.5)	19.7 %
	EVD after index procedure	1633	(16.0)	22.9 %
**Neurosurgical clinical category**				
	General & Trauma	3095	(30.2)	31.9 %
	Oncology	903	(8.8)	28.4 %
	CSF disorders	1771	(17.3)	13.0 %
	Skull base	360	(3.5)	13.6 %
	Neurovascular	4013	(39.2)	29.6 %
	Other	97	(1.0)	21.7 %

### 3.1 Factors associated with 90-day mortality

The results from the model testing the associations between 90-day mortality and the patient characteristics is given in Table 2. A greater risk of death was found to be associated with increasing age, emergency admission, higher RCS CCI, the Neurosurgical clinical category, and EVD timing. Patient sex and deprivation were not associated with mortality. Interactions between age and admission type, Neurosurgical clinical category and EVD timing, and admission type and EVD timing were included in the model.

**Table 2:** Logistic regression model for predicting 90 day post-op mortality.

Patient characteristic		Odds ratio	95 % confidence interval	
Age (years)		1.013	1.010	1.017
*Interaction*	*Age x elective*	1.025	1.011	1.039
Admission type	Non-elective	1.000		
	elective	0.174	0.072	0.420
EVD order	EVD only	1.000		
	EVD before	0.612	0.422	0.887
	EVD same day	0.503	0.418	0.604
	EVD after	0.702	0.533	0.924
*Interaction*	*EVD after x elective*	1.000		
	*EVD after x elective*	1.641	0.61	4.414
RCS Charlson Comorbidity	0	1.000		
Score	1	1.144	1.025	1.278
	2	1.66	1.402	1.880
	3	2.256	1.864	2.991
NSx clinical	General & Trauma	1.000		
category	Oncology	2.867	1.801	4.562
	CSF disorders	0.613	0.420	0.896
	Skull base	0.967	0.364	2.570
	Neurovascular	2.527	2.135	2.991
*Interaction*	*General & Trauma x EVD only*			
	*Oncology x EVD same day*	0.376	0.218	0.647
	*Oncology x EVD before*	0.277	0.136	0.567
	*Oncology x EVD after*	0.400	0.211	0.758
	*Neurovascular x EVD same day*	0.347	0.266	0.454
	*Neurovascular x EVD before*	0.278	0.183	0.423
	*Neurovascular x EVD after*	0.319	0.223	0.457

The model demonstrates the difference in risk for the group of patients who had EVD only compared with the other EVD categories is maintained after accounting for the differences between the groups. The interaction term for clinical category and EVD type captured the difference in mortality for the Oncology and Neurovascular categories associated with the EVD only category compared to when EVD insertion was linked to an index procedure. An interaction term for admission type and EVD type captured the relatively low risk among patients admitted electively for an EVD only procedure compared to the other EVD categories.

The performance of the model was reasonable. The areas under the Receiver Operating Characteristic (ROC) Curve was 0.7105, which equates to a moderate/good discriminative ability. The model was well calibrated (Supplementary Fig. 1) and demonstrated a wide distribution of risk within this patient cohort, with the average predicted risk within the lowest and highest deciles being 8 % and 61 %, respectively.

**Fig. 2:**
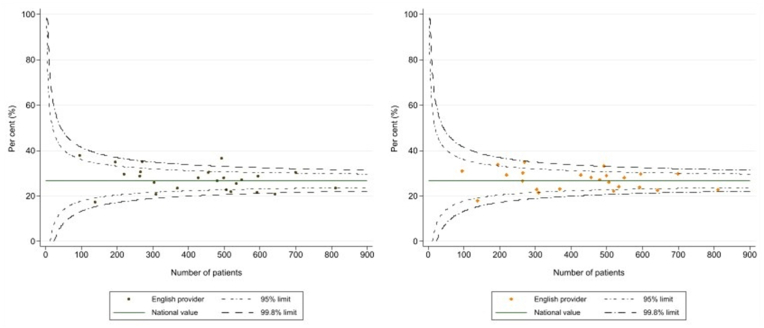
Funnel plots demonstrating mortality within 90 days for all adult Neurosurgical units: Crude morality (left) and risk-adjusted mortality (right).

The funnel plots for crude and adjusted 90-day mortality rates for the 24 adult Neurosurgical centres performing EVD operations is depicted in Fig. 2. The plot of crude rates on the left flags two centres that have values outside the 99.8 % control limits. After adjustment using the logistic regression model, the values for these centres are brought within the outer limits.

### 3.2 30 day readmission, length of stay and unit-level analysis

Table 3 summarises how average length of stay and the proportion of emergency readmissions within 30 days were related to patient characteristics and the type of EVD procedure. For the whole cohort, the median LOS was 21 days (IQR: 10–38), and the 30-day readmission rate was 8.5 %. Age and RCS CCI were not strongly associated with either LOS or the risk of readmission. Patients in the most deprived quintile had a median LOS that was 3 days longer than patients in the least deprived. However, deprivation was not associated with a variation in readmission rates. Elective patients were discharged on average 6 days earlier than emergency patients but the readmission rates were 11.3 % and 8.2 %, respectively.

**Table 3:** Length of stay and readmission as an emergency within 30 days.

		No. of	Length of stay		30-day
Patient characteristic		patients	median	IQR	readmission
Total		10,239	21	(10–38)	8.5 %
Age group (years)					
	18–39	1936	20	(10–38)	11.3 %
	40–49	1654	21	(9–38)	8.2 %
	50–59	2374	22	(11–38)	8.0 %
	60–69	2281	23	(12–41)	7.3 %
	70 +	1994	20	(9–34)	7.8 %
Sex					
	Female	5253	21	(11–36)	8.3 %
	Male	4985	22	(10–39)	8.6 %
Deprivation					
	1 (Most)	2353	23	(11–44)	8.2 %
	2	2149	21	(10–36)	7.9 %
	3	1931	21	(11–36)	8.4 %
	4	1923	21	(10–37)	9.5 %
	5 (Least)	1883	20	(10–35)	8.4 %
Admission type					
	Non-elective	9325	22	(11–38)	8.2 %
	elective	914	16	(7–35)	11.3 %
RCS Charlson Comorbidity Score					
	0	5286	21	(10–37)	7.8 %
	1	3096	23	(11–39)	9.4 %
	2	1243	22	(9–40)	8.6 %
	3	419	20	(9–37)	9.3 %
NSx Clinical Category					
	General & Trauma	3095	20	(9–36)	6.4 %
	Oncology	903	15	(8–30)	13.6 %
	CSF disorders	1771	22	(12–43)	12.9 %
	Skull base	351	21	(11–41)	10.3 %
	Neurovascular	4013	23	(12–38)	6.8 %
	Other	97	34	(15–60)	7.2 %
EVD category					
	EVD only	2954	14	(5–28)	5.5 %
	EVD before	2227	27	(16–45)	9.3 %
	EVD same day	3425	19	(10–34)	9.7 %
	EVD after	1633	32	(19–57)	10.0 %

There was significant variation in these outcomes across Neurosurgical clinical categories. Oncology and CSF disorder patients had shorter LOS but higher readmission rates (13.6 % and 12.9 %, respectively). In contrast, Neurovascular cases had longer stays (23 days) but lower readmission (6.8 %). Furthermore, the EVD categorisation showed differences in both LOS and readmission rates. EVD alone had both the shortest LOS at 14 days (IQR: 5–28) and readmission rate of 5.5 %. This rose to a LOS of 32 days (IQR: 19–57) for the cohort of patients undergoing an EVD procedure after an index procedure and these patients also had the highest readmission rates at 10 %.

Supplementary Fig. 2 shows caterpillar plots for LOS over the different EVD categories by Neurosurgical unit. Generally, the least variation is shown when an EVD is inserted on the same day as an index procedure and there are generally narrower confidence intervals. However, there is variation demonstrated across the other 3 groups. Supplementary Fig. 3 shows a caterpillar plot where the LOS has been adjusted by a quantile regression model. This shows that 7 out of the 24 units have a length of stay where the confidence interval does not include the mean. Even following adjustment, there remains persistent inter-unit variation in median LOS.

**Fig. 3:**
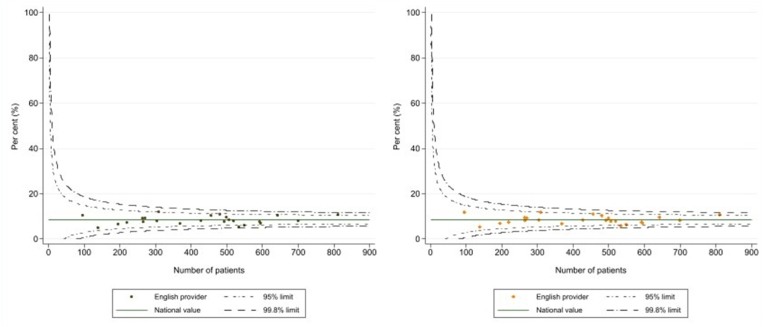
Funnel plots demonstrating readmission within 30 days for all adult Neurosurgical units: Crude (left) and risk-adjusted (right).

Funnel plots for the crude and adjusted 30-day emergency readmission rates are shown in Fig. 3. The plot of adjusted figures on the right side shows a tighter clustering of values around the national average than the plot of unadjusted rates on the left, but there were no units with values outside the 99.8 % control limits on either plot.

## 4 Discussion

In this large national cohort of patients undergoing EVD insertion we found that mortality was high overall (26.7 %), but this varied substantially by both Neurosurgical clinical category and timing of the EVD insertion relative to an index Neurosurgical procedure. The risk adjustment model for mortality narrowed the inter-unit variation. Readmission generally had less variation compared with mortality and the effect of risk-adjustment was less pronounced. For LOS even after risk-adjustment significant variation between units was maintained.

We demonstrate that mortality rises steeply with age as well as RCS CCI. Emergency admissions have a far higher 90-day mortality (27.8 %) compared with elective admissions (15.5 %). However, it should be noted that this figure remains high for an elective Neurosurgical admission (Wahba et al., 2022b). Females have slightly higher mortality across groups than males although this was not statistically significant. General & Trauma, Neurovascular and Oncology patients have the highest mortality rates of 31.9 %, 29.6 % and 28.4 % respectively. LOS was fairly similar across all age groups, comorbidity status and sex. Significant differences were demonstrated among EVD timing with EVD only having a median LOS of 14 days and EVD after an index procedure being 32 days. The lower LOS for EVD-only being no doubt partially explained by the high early mortality. This cohort also had the highest readmission rates at 10 %. Oncology and CSF categories both demonstrated high readmission rates at 13.6 % and 12.9 % respectively. The unit level analysis showed significant variation between centres in terms of LOS. It is of note that both LOS with an overall mean of 21 days (IQR: 10–38) and emergency readmission rates with a mean of 8.5 % are high figures (Ansari et al., 2018; Sander et al., 2020; Vaziri et al., 2014). This suggests that these patients are generally complicated emergency admissions that require significant resources from Neurosurgical centres. This makes it a candidate for quality improvement work within Neurosurgical centres (Madan et al., 2025).

The 2018 prospective, multicentre study of EVD-related infection in the UK placed the 30-day mortality rate at 25.2 % (Jamjoom et al., 2018). We have demonstrated, however, that depending upon how you cohort these patients the mortality can be far higher or indeed lower. This can affect case selection as well as how we counsel patients and their families. Our findings demonstrate that the time at which the EVD is inserted during the patients care pathway and the patient's underlying diagnosis can help predict patient mortality. We would therefore caution against studies that seek to compare the outcomes of EVD procedure as a single cohort without considering these factors. Whilst Neurosurgical audit work can be made more difficult by small numbers we have clearly demonstrated that by not using nuanced cohorts for common procedures and instead simply publishing outcomes for a heterogenous group we risk missing pertinent information within the data (Sibert et al., 2021). These lessons are applicable to other surgical specialties where the same procedure is performed for different reasons. Failure to risk-adjust appropriately or create clinically meaningful cohorts can impact the robustness of findings.

Furthermore, the EVD category of EVD inserted after a Neurosurgical index procedure showed significantly higher LOS, readmission rates and had a longer tail on the Kaplan Meier curve with respect to morality over the first year post-EVD insertion. This cohort of patients may denote the complication of a previous procedure or delayed hydrocephalus, and indeed it has the highest elective admission rate of 20.9 % of all the EVD timing categories. This potentially makes it a candidate to be reported by the National Neurosurgical Audit Programme as a quality measure since the rate of conversion to EVD for different procedures may have a significant impact upon patient outcomes. Further work is needed to elucidate whether outcomes differ based upon specific index procedure in this cohort of patients.

As well as the methodological implications for audit benchmarking and performance monitoring work, our study can also help with decision making for clinicians. The EVD-only group for example, has significantly high 90-day mortality for 70+ year old patients (54.0 %), Oncology patients (58.3 %), Neurovascular patients (59.4 %) and those patients with an RCS CCI of 3+ (60.6 %). The decision to insert an EVD as a life-saving procedure is not always straightforward and we provide evidence that clinicians should be very guarded with respect to the prognosis of patients who undergo this procedure as a first-line one in an emergency setting (Warren et al., 2022; van Donkelaar, 2019). We would welcome further studies from international colleagues to validate these outcome profiles to form a greater consensus that could meaningfully impact patient selection.

There are limitations to consider with respect to our study. Firstly, there may be additional explanatory variables not included in the risk-adjustment model that could improve the performance of the model. This means that further stratification of the patient cohorts by clinical severity is not possible. It was outside of the aims of this paper to fully investigate this. The model itself was internally validated using discrimination and calibration but future work validating it on another cohort would strengthen its use. Our use of LOS as a metric does not incorporate time spent at a secondary hospital recovering from surgery following transfer. Further work is needed to determine whether linking hospital stays as a continuous inpatient stay better stratifies this outcome in Neurosurgical cohorts. Indeed there will be different practices regarding the management of EVDs at different hospitals regarding removal and non-Neurosurgical factors such wait times for gastrostomy may drive variation in outcomes such as LOS independent of the underlying quality of Neurosurgical care. Further work looking into the reasons for variation in LOS, for example, would add greater context to our findings. The use of routinely collected administrative data means that the data may be subject to error in terms of how procedures are classified and whether they are fully captured in HES. The study did not contain a control arm investigating the outcomes of patients with similar pathologies and demographic details who did not undergo the placement of an EVD. Further work such as this may provide greater insights into issues of case selection moving forwards. Furthermore, this study cannot claim to make causal links between the timing of EVD insertion and patient outcome. Indeed our findings instead highlight why more granular data sets are essential if EVD-related outcomes are to be used for performance monitoring in the future.

This novel approach to investigating the outcome of a common Neurosurgical procedure also possesses significant strengths. We present a very large cohort that provides a comprehensive picture of EVD outcomes in routine NHS practice. We introduce a novel, clinically meaningful framework that demonstrates different patient trajectories. By using risk-adjustment to develop funnel and caterpillar plots, the study demonstrates how administrative data can be used to explore variation in EVD outcomes. Our findings highlight clinically relevant subgroups, with “EVD after index procedure” emerging as a potential signal of complications in the patient pathway. However, in their current form EVD-related outcomes are best suited to hypothesis-generation and further work is needed before a complex cohort such as this could be used for formal benchmarking.

## 5 Conclusion

Our findings demonstrate that treating all EVD insertions as a single cohort obscures clinically meaningful differences in patient trajectories and leads to misleading comparisons of outcomes. Stratifying cases by the timing of the EVD relative to the index neurosurgical procedure reveals marked variation in outcome profiles and substantially improves the performance of risk-adjustment models. Although the absence of detailed severity markers in administrative data means that conclusions about quality of care must be interpreted cautiously, this study illustrates how carefully constructed, clinically meaningful cohorts can transform the interpretation of common neurosurgical procedures. The same methodological approach can be applied in less complex, more uniform patient groups to support fair and robust benchmarking across neurosurgical centres.

**Ethics approval:** The study is exempt from UK National Research Ethics Service (NRES) approval because it involved the analysis of routinely collected, anonymised data. HES data were made available by NHS Digital (Copyright, 2018, Reused with the permission of NHS Digital. All rights reserved). Approvals for the use of anonymised HES data were obtained as part of the standard NHS Digital data access process. The data are routinely collected by the National Health Service (NHS) and anonymised at source. The data are obtained as

part of the standard NHS Digital data access process. NHS Digital has a legal obligation to collect these data and it does not require the consent of individual patients. Please see here:https://digital.nhs.uk/about-nhs-digital/our-work/keeping-patient-data-safe/gdpr/gdpr-register/hospital-episode-statistics-gdpr/hospital-episode-statistics-hes-gdpr-information.

## Funding sources

DT is the National Neurosurgical Audit Programme Fellow, a role wholly funded by the Society of British Neurological Surgeons. AH is supported by the Royal College of Surgeons of England and the Cambridge NIHR Biomedical Research Centre. PH is supported by the UK NIHR (Senior Investigator Award, Cambridge Biomedical Research Centre, Brain Injury Medtech Co-operative, Global Health Research Group on Acquired Brain and Spine Injury) and the Royal College of Surgeons of England.

## Declaration of competing interest

The authors declare the following financial interests/personal relationships which may be considered as potential competing interests: Daniel Thompson reports financial support was provided by Society of British Neurological Surgeons. If there are other authors, they declare that they have no known competing financial interests or personal relationships that could have appeared to influence the work reported in this paper.

## Appendix 3

### Omitted OPCS-4 procedure codes

A201 – Drainage of ventricle of brain NEC.

A208 – Other specified other operations on ventricle of brain.

A113 – Monitoring of pressure in tissue of brain.

A203 – Monitoring of pressure in ventricle of brain.

A535 – Drainage of cerebrospinal fluid NEC.

A538 – Other specified drainage of spinal canal.

A539 – Unspecified drainage of spinal canal.

A522 – Therapeutic sacral epidural injection.

A651 – Carpal tunnel release.

A521 – Therapeutic lumbar epidural injection.

A671 – Cubital tunnel release.

A528 – Other specified therapeutic epidural injection.

A523 – Epidural blood patch.

A529 – Unspecified therapeutic epidural injection.

## Appendix A Supplementary data

Fig. S1 Hosmer-Lemeshow plot depicting the calibration of the final logistic regression model. Fig. S1

Fig. S2 Caterpillar plots of LOS by EVD category to compare the unadjusted median LOS of Neurosurgical units. Fig. S2

Fig. S3 Caterpillar plot to compare the adjusted LOS of Neurosurgical units. Fig. S3
