# Supplementary material for: Patterns of care and outcomes following external ventricular drain placement: Insights from the England HES administrative data set
Source: Brain Spine. 2025 Dec 16;6:105906. doi: 10.1016/j.bas.2025.105906 (PMC12771326; doi:10.1016/j.bas.2025.105906)
Supplement: Fig. S1 — Hosmer-Lemeshow plot depicting the calibration of the final logistic regression model. [file mmc1.docx]

**Figure S1: Hosmer-Lemeshow plot depicting the calibration of the final logistic regression model**

**
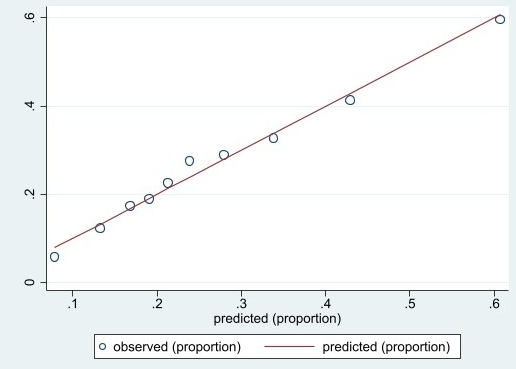
**
