# Supplementary material for: Patterns of care and outcomes following external ventricular drain placement: Insights from the England HES administrative data set
Source: Brain Spine. 2025 Dec 16;6:105906. doi: 10.1016/j.bas.2025.105906 (PMC12771326; doi:10.1016/j.bas.2025.105906)
Supplement: Fig. S2 — Caterpillar plots of LOS by EVD category to compare the unadjusted median LOS of Neurosurgical units. [file mmc2.docx]

**
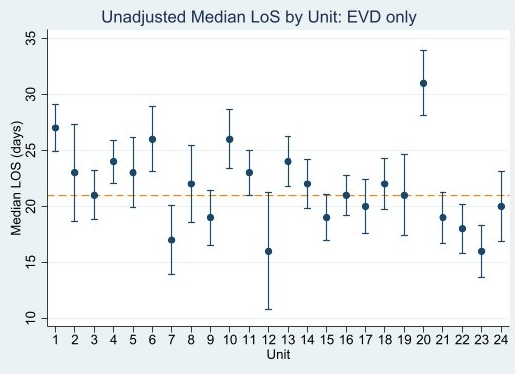
**

**
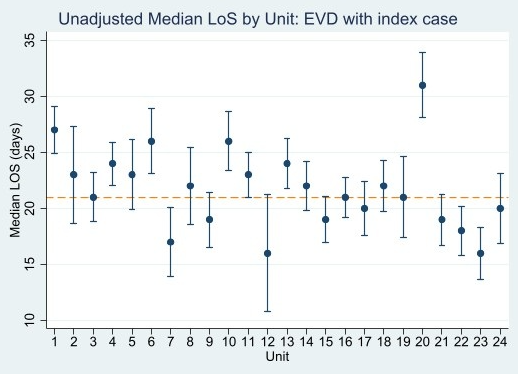
**
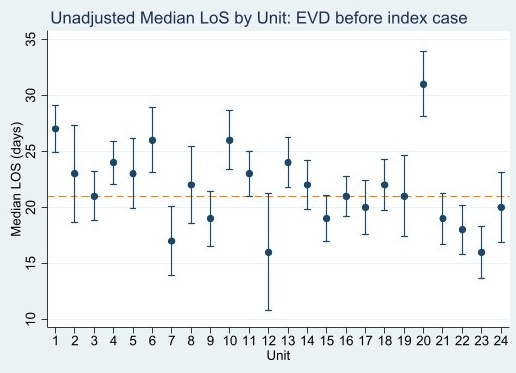


**
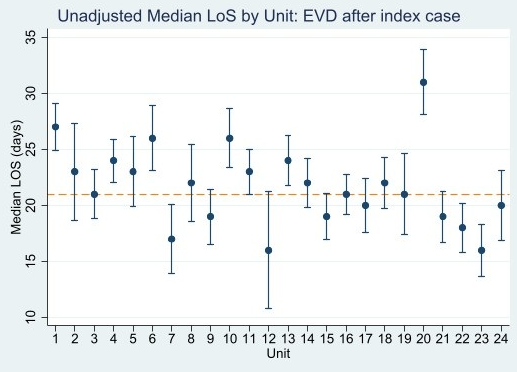
Figure S2: Caterpillar plots of LOS by EVD category to compare the unadjusted median LOS of Neurosurgical units**
