# Supplementary figures and images for: Patterns of care and outcomes following external ventricular drain placement: Insights from the England HES administrative data set
Source: Brain Spine. 2025 Dec 16;6:105906. doi: 10.1016/j.bas.2025.105906 (PMC12771326; doi:10.1016/j.bas.2025.105906)

**Figure S3: Caterpillar plot to compare the adjusted LOS of Neurosurgical units**

**
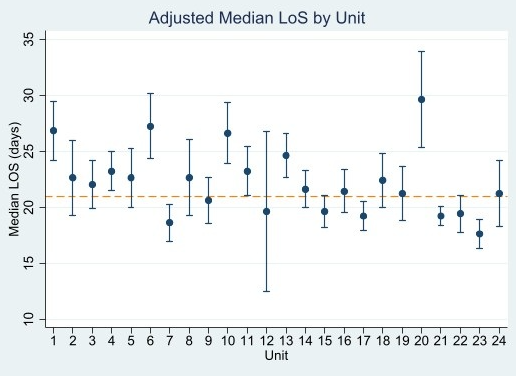
**

Supplement: Fig. S3 — Caterpillar plot to compare the adjusted LOS of Neurosurgical units. [file mmc3.docx]
