# Supplementary material for: Patterns of care and outcomes following external ventricular drain placement: Insights from the England HES administrative data set
Source: Brain Spine. 2025 Dec 16;6:105906. doi: 10.1016/j.bas.2025.105906 (PMC12771326; doi:10.1016/j.bas.2025.105906)
Supplement: Multimedia component 4 [file mmc4.docx]

**Table S1: Descriptive statistics categorised by EVD category using a Chi2 test of independence to compare demographic details across EVD category**

|  |  | **EVD category** | | | | | | | | | | | |
| --- | --- | --- | --- | --- | --- | --- | --- | --- | --- | --- | --- | --- | --- |
|  |  | **EVD only** | | | **EVD before index procedure** | | | **EVD with index procedure** | | | **EVD after index procedure** | | |
|  |  | **No. of patients** | | **90-day mortality** | **No. of patients** | | **90-day mortality** | **No. of patients** | | **90-day mortality** | **No. of patients** | | **90-day mortality** |
| **Total** |  | 2,954 | (%) | 43.20% | 2,227 | (%) | 18.30% | 3,425 | (%) | 19.70% | 1,633 | (%) | 22.90% |
| **Age at admission (years)** | | |  |  |  |  |  |  |  |  |  |  |  |
|  | **16-39** | 474 | 16.1% | 32.5% | 383 | 17.2% | 9.1% | 702 | 20.5% | 11.8% | 377 | 23.1% | 15.7% |
|  | **40-49** | 450 | 15.2% | 40.7% | 366 | 16.4% | 20.0% | 567 | 16.6% | 17.3% | 271 | 16.6% | 20.3% |
|  | **50-59** | 691 | 23.4% | 41.80% | 537 | 24.1% | 14.5% | 763 | 22.3% | 17.2% | 383 | 23.5% | 23.0% |
|  | **60-69** | 648 | 21.9% | 42.8% | 559 | 25.1% | 21.8% | 725 | 21.2% | 25.5% | 349 | 21.4% | 26.7% |
|  | **70 +** | 691 | 23.4% | 54.0% | 382 | 17.2% | 25.9% | 668 | 19.5% | 26.5% | 253 | 15.5% | 31.2% |
| **Sex** |  |  |  |  |  |  |  |  |  |  |  |  |  |
|  | **Female** | 1,268 | 42.90% | 47.40% | 1,271 | 57.10% | 18.30% | 1,851 | 54.00% | 19.30% | 863 | 52.90% | 22.80% |
|  | **Male** | 1,686 | 57.10% | 40.00% | 956 | 42.90% | 18.30% | 1,574 | 46.00% | 20.10% | 769 | 47.10% | 22.90% |
| **Deprivation** | |  |  |  |  |  |  |  |  |  |  |  |  |
|  | **1 (Most)** | 672 | 22.80% | 42.70% | 502 | 22.50% | 18.70% | 765 | 22.30% | 19.50% | 414 | 25.40% | 24.20% |
|  | **2** | 645 | 21.80% | 45.70% | 459 | 20.60% | 18.70% | 724 | 21.10% | 21.70% | 321 | 19.70% | 23.40% |
|  | **3** | 534 | 18.10% | 42.70% | 454 | 20.40% | 15.60% | 645 | 18.80% | 18.80% | 298 | 18.30% | 23.50% |
|  | **4** | 574 | 19.40% | 44.40% | 392 | 17.60% | 17.90% | 638 | 18.60% | 19.10% | 319 | 19.50% | 19.40% |
|  | **5 (Least)** | 529 | 17.90% | 39.90% | 420 | 18.90% | 20.50% | 653 | 19.10% | 19.10% | 281 | 17.20% | 23.80% |
| **Admission type** | |  |  |  |  |  |  |  |  |  |  |  |  |
|  | **Non-elective** | 2,851 | 96.50% | 44.40% | 2,183 | 98.00% | 18.40% | 3,000 | 87.60% | 20.60% | 1,291 | 79.10% | 23.60% |
|  | **Elective** | 103 | 3.50% | 8.70% | 44 | 2.00% | 13.60% | 425 | 12.40% | 13.40% | 342 | 20.90% | 20.50% |
| **RCS Charlson Comorbidity Score** | | | | |  |  |  |  |  |  |  |  |  |
|  | **0** | 1,552 | 52.50% | 40.10% | 1,243 | 55.80% | 14.80% | 1,727 | 50.40% | 15.50% | 764 | 46.80% | 20.30% |
|  | **1** | 853 | 28.90% | 42.00% | 642 | 28.80% | 18.20% | 1,064 | 31.20% | 20.00% | 537 | 32.90% | 21.60% |
|  | **2** | 361 | 12.20% | 50.10% | 228 | 10.20% | 26.30% | 426 | 12.40% | 29.30% | 228 | 14.00% | 29.00% |
|  | **3** | 188 | 6.40% | 60.60% | 114 | 5.10% | 40.40% | 208 | 6.10% | 32.70% | 104 | 6.40% | 35.60% |
| **Neurosurgical clinical category** | | | | |  |  |  |  |  |  |  |  |  |
|  | **General & Trauma** | 1,715 | 58.1% | 37.5% | 170 | 7.60% | 24.10% | 902 | 26.30% | 24.40% | 308 | 18.90% | 27.30% |
|  | **Oncology** | 96 | 3.3% | 58.3% | 139 | 6.20% | 21.60% | 469 | 13.70% | 22.40% | 199 | 12.20% | 32.20% |
|  | **CSF disorders** | 223 | 7.6% | 17.5% | 588 | 26.40% | 12.60% | 627 | 18.30% | 12.10% | 333 | 20.40% | 12.30% |
|  | **Skull base** | 23 | 0.8% | 26.1% | 36 | 1.60% | 5.60% | 167 | 4.90% | 10.20% | 134 | 8.20% | 17.90% |
|  | **Vascular** | 886 | 30.0% | 59.4% | 1,279 | 57.40% | 20.30% | 1,241 | 36.20% | 20.40% | 607 | 37.20% | 24.60% |
|  | **Other** | 11 | 0.4% | 54.6% | 15 | 0.70% | 0.00% | 19 | 0.60% | 15.80% | 52 | 3.20% | 23.10% |
