# Supplementary material for: Patterns of care and outcomes following external ventricular drain placement: Insights from the England HES administrative data set
Source: Brain Spine. 2025 Dec 16;6:105906. doi: 10.1016/j.bas.2025.105906 (PMC12771326; doi:10.1016/j.bas.2025.105906)
Supplement: Multimedia component 7 [file mmc7.docx]

**Appendix 3**

**Omitted OPCS-4 procedure codes**

A201 – Drainage of ventricle of brain NEC

A208 – Other specified other operations on ventricle of brain

A113 – Monitoring of pressure in tissue of brain

A203 – Monitoring of pressure in ventricle of brain

A535 – Drainage of cerebrospinal fluid NEC

A538 – Other specified drainage of spinal canal

A539 – Unspecified drainage of spinal canal

A522 – Therapeutic sacral epidural injection

A651 – Carpal tunnel release

A521 – Therapeutic lumbar epidural injection

A671 – Cubital tunnel release

A528 – Other specified therapeutic epidural injection

A523 – Epidural blood patch

A529 – Unspecified therapeutic epidural injection
